# Effect of vagus nerve stimulation for the treatment of drug-resistant epilepsy: A protocol of systematic review and meta-analysis

**DOI:** 10.1097/MD.0000000000020315

**Published:** 2020-06-05

**Authors:** Peng Chen, Mei-mei Hao, Jiang Zhu, Zeng-ye Yang

**Affiliations:** a Department of Neurology, The First Hospital of Yulin, Yulin; b Department of Neurology, Yan’an People's Hospital, Yan’an, China.

**Keywords:** drug-resistant epilepsy, effect, safety, vagus nerve stimulation

## Abstract

**Background:**

Drug-resistant epilepsy (DRE) is a very tricky disorder, which greatly affects quality of life in such patients. Relevant studies suggested that vagus nerve stimulation (VNS) has potential benefits for DRE. However, there are inconsistent conclusions. The purpose of this study is to investigate whether VNS is effective and safety for DRE.

**Methods:**

To collect comprehensive randomized controlled trials (RCTs), the following electronic databases will be retrieved: MEDLINE, EMBASE, Cochrane Library, Web of Science, PsycINFO, CINAHL, AMED, and China National Knowledge Infrastructure from the commencement of each electronic database up to the present with no language restrictions. Two authors will independently carry out all procedures of literature selection, information collection, and risk of bias assessment. Any objections will be worked out by a third author through consultation. The risk of bias for each included trial will be identified using Cochrane risk of bias tool, and statistical analysis will be performed utilizing RevMan 5.3 software.

**Results:**

This study will synthesize the data from the present eligible high quality RCTs to assess whether VNS is effective and safety for DRE.

**Conclusion:**

This study will provide systematic evidence of VNS for the treatment of patients with DRE.

**Systematic review registration:**

INPLASY202040086.

## 1 Introduction

Epilepsy is a very frequent neurological disorder, which characterized by the presence of spontaneous and recurrent seizures.^[1–5]^ It is reported that about 50 million people suffer epilepsy around the world.^[6]^ Its prevalence varies from 0.5% to 1% of general population in the developed countries.^[7,8]^ Of those, there are about 30% patients who experience drug-resistant epilepsy (DRE).^[6,9]^ A variety of studies have reported that vagus nerve stimulation (VNS) can be used to treat DRE.^[10–21]^ However, no systematic review investigated its efficacy, and its results are still inconsistent. Thus, the present study will aim to assess the effect and safety of VNS for the treatment of DRE.

## 2 Methods and analysis

### 2.1 Study registration

This study has been registered on INPLASY202040086. It is reported strictly according to the Preferred Reporting Items for Systematic Reviews and Meta-Analyses Protocols guideline.

### 2.2 Eligibility criteria

#### 2.2.1 Types of studies

We will include randomized controlled trials (RCTs) of VNS therapy for patients with DRE. However, we will exclude studies that belong to the case report, case series, review, comment, uncontrolled trials, non-RCTs, and quasi-RCTs. No language and publication status limitations will be applied.

#### 2.2.2 Types of participants

We will consider all adult patients (18 years old or over) who were diagnosed as DRE. There is no restriction of race, sex, country, educational background, and economic status.

#### 2.2.3 Types of interventions

This study will include trials that used VNS therapy alone in the intervention group.

The control group can use any management for patients with DRE. However, we will not consider combination therapy of VNS and other therapies.

#### 2.2.4 Types of outcome measurements

The primary outcome is seizure freedom. Secondary outcomes are frequency of seizures, quality of life, all cause mortality, visits to the emergency room, and any expected or unexpected adverse events.

### 2.3 Search methods for the identification of studies

#### 2.3.1 Electronic database searches

The following electronic databases will be sought from the commencement up to the present with no language and publication status restrictions: MEDLINE, EMBASE, Cochrane Library, Web of Science, PsycINFO, CINAHL, AMED, and China National Knowledge Infrastructure. We will include any RCTs that investigated the effect and safety of VNS for the treatment of patients with DRE. Take MEDLINE as an example, the specific search strategy is stated in Table 1. The similar search strategies will be modified and will be applied to the other electronic databases.

**Table 1:**
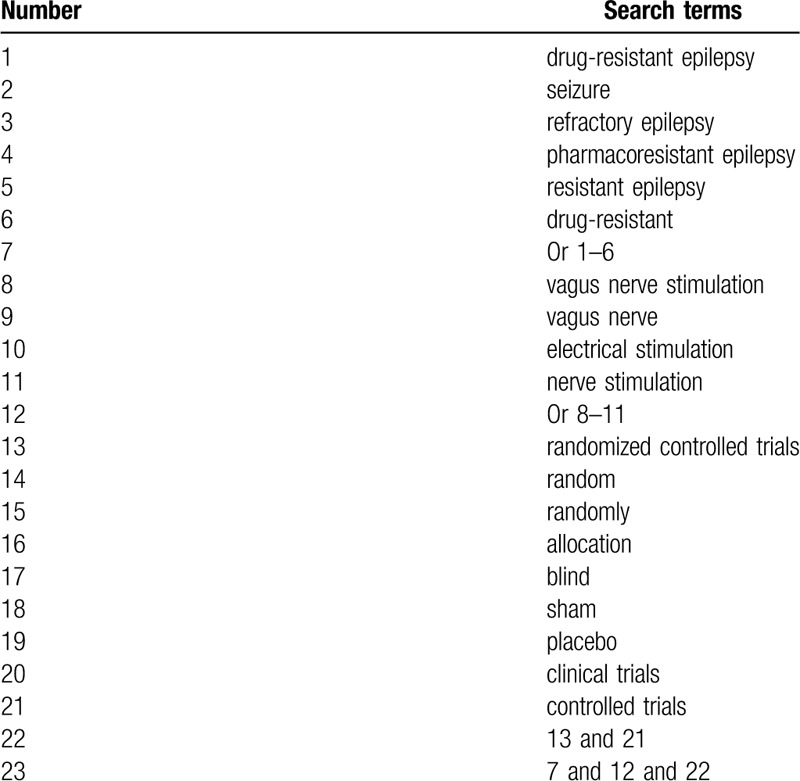
Search strategy of MEDLINE.

#### 2.3.2 Other resources searches

Aside from above electronic databases, we will review and identify reference lists of relevant reviews, conference abstracts, dissertations, and websites of clinical trials registry.

### 2.4 Data collection and analysis

#### 2.4.1 Selection of studies

All retrieved literatures will be imported to the Endnote 9.1 to remove any duplicates. Two authors will independently screen the titles/abstracts of all searched records, and unrelated studies will be excluded. After that, full texts of remaining trials will be read carefully as a second filtration. Two authors will crosscheck the included trials. Any different views on the selection of studies will be solved by a third author through discussion. The detailed selection process will be presented in a flow chart.

#### 2.4.2 Data extraction

Two authors will independently collect data to fill out the pre-designed data extraction sheet. If any disagreements occur, a third author will be involved to settle down such issues through discussion. The extracted information consists of tile, first author, country, year of publication, methodological quality, patient characteristics, details of intervention and controls, outcomes, results and findings, follow-up, adverse events, funding sources, and conflict of interest.

#### 2.4.3 Risk of bias assessment

Based on the guideline of the Cochrane Handbook of Systematic Reviews of Interventions, 2 authors will independently assess the risk of bias for each included trial. We will appraise through 7 aspects, and each one will be rated into 3 levels: low, unclear, and high risk of bias. Any divergences between 2 authors will be solved by a third author through consultation.

#### 2.4.4 Dealing with missing data

When there is missing or insufficient data, the related corresponding authors will be contacted to obtain it. If we cannot receive such data, we will analyze the data at hand, and will discuss its potential impacts as a limitation.

#### 2.4.5 Data synthesis

RevMan 5.3 (Cochrane Community; London, UK) software will be utilized to perform all data analysis and to carry out a meta-analysis if it is possible. For continuous outcomes (e.g., seizure freedom), we will present them as mean difference or standardized mean difference with 95% confidence intervals (CIs). For dichotomous outcomes (e.g., all cause mortality), we will calculate them as risk ratio and 95% CIs. We will use *I*^2^ statistic to investigate the heterogeneity across the eligible trials. If the values of *I*^2^ are ≤50%, reasonable heterogeneity will be considered, and a fixed-effects model will be employed. Meanwhile, we will undertake meta-analysis if sufficient similar studies in relation to the study information, participant characteristics, interventions, comparators, and outcomes. On the other hand, if the values of *I*^2^ are >50%, substantial heterogeneity will be regarded, and a random-effect model will be exerted. At the same time, we will implement subgroup analysis to identify possible sources for the significant heterogeneity.

#### 2.4.6 Assessment of reporting bias

If the quantify of eligible trials is over 10, we will perform funnel plot and Egger regression test to assess the potential publication bias.^[22]^

#### 2.4.7 Subgroup analysis

Subgroup analysis will be carried out based on the different types of interventions, comparators, and outcome measurements.

#### 2.4.8 Sensitivity analysis

We will undertake sensitivity analysis to assess the robustness of results by removing high risk of bias studies when significant heterogeneity exists.

### 2.5 Grading the quality of evidence

We will appraise the quality of evidence for each outcome using Grading of Recommendations Assessment, Development, and Evaluation^[23]^ through 5 domains. Each one is graded the quality into 4 levels (very low, low, moderate, and high).

### 2.6 Dissemination and ethics

This study will be published through a peer-reviewed scientific journal. No formal ethical approval is required, because no individual patient data will be obtained.

## 3 Discussion

To our best knowledge, this is the first study to explore the effect and safety of VNS for the treatment of patients with DRE. It will attempt to conduct a comprehensive search and systematic analysis of the existing evidence to fill this gap in the research field. Its findings will supply a detailed summary of the present evidence of VNS for the treatment of patients with DRE. It may provide guidance and reference for clinical practice, future research, as well as health-related policy maker.

## Author contributions

**Conceptualization:** Peng Chen, Mei-mei Hao, Jiang Zhu, Zeng-ye Yang.

**Data curation:** Peng Chen, Mei-mei Hao, Zeng-ye Yang.

**Formal analysis:** Jiang Zhu, Zeng-ye Yang.

**Funding acquisition:** Mei-mei Hao.

**Investigation:** Mei-mei Hao.

**Methodology:** Peng Chen, Jiang Zhu, Zeng-ye Yang.

**Project administration:** Mei-mei Hao.

**Resources:** Peng Chen, Jiang Zhu, Zeng-ye Yang.

**Software:** Jiang Zhu, Zeng-ye Yang.

**Supervision:** Peng Chen, Mei-mei Hao.

**Validation:** Mei-mei Hao, Jiang Zhu, Zeng-ye Yang.

**Visualization:** Peng Chen, Mei-mei Hao, Zeng-ye Yang.

**Writing – original draft:** Peng Chen, Mei-mei Hao, Jiang Zhu, Zeng-ye Yang.

**Writing – review & editing:** Peng Chen, Mei-mei Hao, Zeng-ye Yang.
